# Progress in research on the role of fibrinogen in lung cancer

**DOI:** 10.1515/biol-2020-0035

**Published:** 2020-06-05

**Authors:** Xing Liu, Bin Shi

**Affiliations:** Department of Respiratory Medicine, The Affiliated Suqian Hospital of Xuzhou Medical University, Suqian 223800, Jiangsu, China

**Keywords:** fibrinogen, cancer biomarkers, lung cancer

## Abstract

Lung cancer is one of the most prevalent malignancies worldwide. Local recurrence and distant metastasis remain the major causes of treatment failure. It has been recognized that the process of tumor growth and metastasis involves multiple interactions between tumor and host. Various biomarkers have been used for predicting tumor recurrence, metastasis, and prognosis in patients with lung cancer. However, these biomarkers are still controversial and require further validation. The relationship between malignancy and coagulation system disorders has been explored for more than a century. Fibrinogen is the most abundant plasma coagulation factor synthesized mainly by hepatic cells. Increased plasma fibrinogen levels were observed in various carcinomas such as gastric cancer, colon cancer, and pancreatic cancer. Recent studies have also investigated the role of fibrinogen in patients with lung cancer. This review aimed to address the role of fibrinogen in lung cancer.

## 1 Introduction

Lung cancer is a leading cause of cancer death around the world. The 5-year survival rate is only 15%, although various therapies are adopted [1]. Local recurrence and distant metastasis are the main reasons for treatment failure. Numerous factors including carcinoembryonic antigen, neuron-specific enolase, cytokeratin-19 fragments (CYFRA 21-1), and D-dimer for early diagnosis of distant metastasis, tumor recurrence, and prognosis in patients with lung cancer have been intensively identified [2,3]. However, the majority of these conventional indicators are still unsatisfactory.

Tumor-induced coagulation system disorders are commonly implicated in patients with malignancies. Fibrinogen is an essential constituent of the coagulation system. It is mainly synthesized in the liver and released into the circulation in response to systemic inflammation and malignancy. An increasing body of evidence has indicated the association between fibrinogen and tumor clinical stage, angiogenesis, metastatic spread, and response to therapy in patients with cancer [4,5]. The clinical value of fibrinogen in patients with lung cancer has also been investigated previously. To further understand its role, we reviewed fibrinogen estimation in patients with lung cancer.

## 2 Fibrinogen expression in lung cancer

High levels of fibrinogen have been observed in patients with lung cancer (Table 1). Kim et al. [9] reported that serum fibrinogen levels were increased in about 55.2% of patients with advanced non-small cell lung cancer (NSCLC), and its concentration may be higher in patients with squamous cell carcinoma than in patients with adenocarcinoma (4.5 ± 0.13 g/L vs 3.6 ± 0.28 g/L, *p* = 0.008) [4]. Elevated serum fibrinogen concentration can change blood viscosity, rheology, and endothelial function, which may facilitate microthrombosis in the pulmonary capillaries and make patients vulnerable to inflammation and ischemia [10,11]. Therefore, detection of serum fibrinogen levels may reflect lung tissue destruction. As previously reported, mean plasma fibrinogen concentration was associated with the pathological T stage but not with the pathological N stage [4]. However, other investigators found that there were no statistically significant differences in fibrinogen levels according to the TNM stage [9].

**Table 1:** The expression of fibrinogen in lung cancer

Histology (*n*)	*n*	Parameters evaluated	Main outcome of the study	Ref.
AC (18), SQ (69), LCC (2), other NSCLC (3)	93	Fibrinogen	Elevated plasma fibrinogen level was positively correlated with increasing tumor size, advanced pathological T stage, and SCC.	[4]
NS	55	Fibrinogen and FDP	Serum fibrinogen and FDP levels were elevated in lung cancer patients with metastases.	[6]
AC (165), SQ (106), LCC (22), SCC (46)	339	Fibrinogen	Increased serum fibrinogen levels were associated with advanced SCC or LCC.	[7]
AC (114), SQ (61), other NSCLC (9)	184	Fibrinogen	High pre- and postoperative fibrinogen levels in patients with NSCLC were associated with lymph node involvement of the tumor and pathologic stages.	[8]

AC, adenocarcinoma; FDP, fibrinogen degradation product; LCC, large cell carcinoma; *n*, number of specimens; NS, not specified; NSCLC, non-small cell lung cancer; SCC, small cell carcinoma; SQ, squamous cell lung cancer.

Guan et al. [12] explored the relationship between fibrinogen and epidermal growth factor receptor (EGFR) mutation status in patients with NSCLC; they found that plasma fibrinogen levels were distinctly higher in patients with wild-type EGFR gene than in patients with EGFR mutations (3.57 g/L vs 2.95 g/L, *p* < 0.001). This shows that fibrinogen may assist in predicting EGFR mutation status in patients with NSCLC. However, additional studies are required to fully illustrate the molecular mechanism between fibrinogen and EGFR gene mutation status.

## 3 Role of fibrinogen in tumor progression

Cancer-related coagulation disorders have been thought to be responsible for the tumor cell growth, invasion, and distant metastasis. Recently, a series of experiments have shown the significance of fibrinogen in the pathogenesis of the cancer process [13,14,15]. As tumor cells dissociate from the primary focus into the circulation, fibrinogen can serve as a scaffold to bind tumor cells and platelets [15], which in turn contributes to tumor cell adherence to the distant organ and promotes tumor angiogenesis. Furthermore, fibrinogen has been implicated in the formation of tumor stroma, which provides gas exchange and nutrient for rapidly proliferating tumor cells.

Palumbo et al. [16] demonstrated that the deficiency of fibrinogen strongly reduced the spontaneous metastatic potential through both hematogenous and lymphatic routes but has no influences on the time required for the formation of palpable tumors, angiogenesis, and overall tumor architecture. Similarly, Palumbo et al. [17] also found that fibrinogen deficiency dramatically reduced the incidence of pulmonary metastases in fibrinogen-deficient transgenic mice. However, additional experiments are required to fully illustrate the relationship between fibrinogen and tumor metastasis.

As an extracellular protein matrix, fibrinogen not only promotes the migration of tumor cells but also protects tumor cells from the innate immune surveillance systems [14]. Zheng et al. [18] reported that fibrinogen can protect tumor cells from natural killer (NK) cell-mediated cytotoxicity by accumulating and forming dense fibrin layers around tumor cells. Gropp et al. [6] also found that fibrinogen split products can suppress immune reactions. Therefore, we can make the treatment more effective for patients with cancer by reducing the activity of the coagulation system.

## 4 Fibrinogen and the prognosis of lung cancer

The potential predictive value of fibrinogen for prognosis in patients with lung cancer has been reported in the previous investigations (Table 2). A significant relationship between initial response to chemotherapy and decreased fibrinogen levels was found in patients with elevated plasma levels of fibrinogen [5]. Jones et al. [4] reported that high fibrinogen values (plasma fibrinogen >5 g/L) were related to incomplete resection in patients with NSCLC. A retrospective analysis explored the correlation between preoperative serum fibrinogen levels and postoperative pulmonary complications (PPCs) in patients who underwent lung cancer resection. They found that high levels of preoperative serum fibrinogen (>400 mg/dL) were associated with the increased risk of PPCs, and it was an independent predictor regardless of sex and smoking status [30]. Jiang et al. [8] evaluated the value of pre- and postoperative plasma fibrinogen levels in predicting tumor recurrence and metastasis in 184 patients with I–IIIA NSCLC, who underwent radical surgery. They found a correlation between high pre- and postoperative plasma fibrinogen levels and tumor recurrence. This indicated that plasma fibrinogen levels may be used to predict tumor metastasis and recurrence for lung cancer patients who underwent surgery.

**Table 2:** Fibrinogen and the prognosis of lung cancer

Parameters evaluated	Main outcome of the study	Ref.
Fibrinogen	High fibrinogen values were associated with incomplete resection in NSCLC.	[4]
Fibrinogen	The reduction of plasma fibrinogen levels can evaluate the efficacy of chemotherapy in advanced NSCLC.	[5]
Fibrinogen	Pre- and postoperative plasma fibrinogen levels can predict tumor recurrence and metastasis for patients with NSCLC.	[8]
Fibrinogen	High fibrinogen levels were associated with poor prognosis in patients with advanced NSCLC.	[9]
Fibrinogen	Preoperative serum fibrinogen level was an independent prognostic marker in patients with operable NSCLC.	[19]
Fibrinogen	High plasma fibrinogen concentration indicated poor prognosis for NSCLC patients with brain metastases.	[20]
Fibrinogen	Plasma fibrinogen can predict the chemotherapy efficacy for patients with SCLC.	[21]
Fibrinogen, NLR	Plasma fibrinogen and NLR may predict the prognosis of patients with resectable NSCLC.	[22]
FA score	The FA score may be a promising prognostic predictor in patients with NSCLC.	[23]
AFR	High pre-resection AFR was associated with longer OS and disease-free survival (DFS) of patients with NSCLC.	[24]
Fibrinogen, AFR	AFR and fibrinogen were promising biomarkers to predict the clinical outcome of patients with NSCLC.	[25]
Fibrinogen, NLR	Combined fibrinogen and NLR can be used to predict prognosis for patients with resectable early-stage NSCLC.	[26]
AFR	Pretreatment AFR can predict the survival of patients with advanced NSCLC, who underwent first-line platinum-based chemotherapy.	[27]
Fibrinogen	Fibrinogen levels may influence the PFS of patients with NSCLC.	[28]
Fibrinogen, NLR	Combination of fibrinogen and NLR can predict tumor progression and the postoperative survival of patients with NSCLC.	[29]

AFR, albumin/fibrinogen ratio; FA score, fibrinogen and serum albumin levels; NLR, neutrophil-to-lymphocyte ratio; NSCLC, non–small cell lung cancer; SCLC, small cell lung cancer.

Several investigators reported that patients with higher plasma fibrinogen levels tend to have poor progression-free survival (PFS) and overall survival (OS) [19,21]. A meta-analysis suggested that the level of plasma fibrinogen ≥400 mg/dL could be a promising indicator for worse OS in patients with lung cancer [31]. Ying et al. [27] explored the usefulness of the albumin-to-fibrinogen ratio (AFR) in patients with advanced NSCLC; they found that the PFS and OS in the high AFR group (>8.02) were markedly improved compared with those in the low AFR group. Therefore, fibrinogen may serve as a candidate biomarker for disease monitoring and prognostic evaluation in patients with lung cancer.

## 5 Fibrinogen and the treatment of lung cancer

It is increasingly recognized that the variation in fibrinogen concentration is associated with the treatment of malignancies. Zhu et al. [21] showed that fibrinogen levels decreased after one and two chemotherapy cycles in patients with small cell lung cancer (SCLC), and the degree of decline was associated with the treatment response. Morano et al. [32] also revealed that serum fibrinogen levels were significantly reduced after 4 weeks of pulmonary rehabilitation in patients with lung cancer.

Some investigators have focused on the therapeutic strategies for controlling tumor metastasis by improving the hypercoagulable status. As reported in the previous studies, the inhibition of tissue factor, factor Xa, and thrombin significantly reduced the experimental hematogenous metastasis [33]. Palumbo et al. [17] also found that thrombin inhibitor substantially diminished the metastatic potential of circulating tumor cells in fibrinogen-deficient mice but had no apparent influence on tumor cell proliferation *in vitro*. Therefore, anticoagulation therapy may prolong the survival time of patients with cancer.

## 6 Conclusion

The treatment and prognosis of cancer depend on the tumor stage, presence of comorbidities, and other factors. Various biomarkers and their combinations for tumor progression and prognosis have been explored. The coagulation abnormality in patients with cancer has been recognized for decades. Considerable attention has been paid to the relationship between the progression of lung cancer and fibrinogen. As we have mentioned above, fibrinogen is a valuable biomarker to predict tumor status, EGFR mutation status, and clinical outcome in patients with lung cancer and is associated with tumor progression and metastasis. As a noninvasive method, measurement of fibrinogen is simple, economical, and less traumatic in clinical practice. Further studies are needed to elucidate the relationship between fibrinogen and lung cancer biology.
